# A Case Report of a Massive Epignathus

**DOI:** 10.25122/jml-2019-0164

**Published:** 2020

**Authors:** Farhad Naleini, Nazanin Farshchian, Mahmood Mehrbakhsh, Parisa Bahrami Kamangar

**Affiliations:** 1. Department of Radiology, Clinical Research Development Center, Kermanshah University of Medical Sciences, Kermanshah, Iran; 2. Kermanshah University of Medical Sciences, Kermanshah, Iran

**Keywords:** Prenatal diagnosis, epignathus, teratoma

## Abstract

Epignathus is a rare congenital orofacial teratoma. Teratomas are tumors that originate from all three germs cell layers. Tumor size is an important prognostic factor, and we describe the case of massive epignathus identified by sonography at 25 weeks. Our case was a 35-year-old pregnant woman that was subjected to a routine ultrasound at 25 weeks of gestation, and epignathus was diagnosed. Labor pain started in the 28th week of the pregnancy, so the dead fetus was aborted, and curettage was conducted. A pathologic sample was sent to the laboratory, and benign teratoma was diagnosed. Because fetal epignathus has a wide range of outcomes, early prenatal diagnosis is essential for optimal management.

## Introduction

Head and neck tumors are rare. Epignathus is a rare congenital orofacial teratoma of Rathke’s pouch, and it usually appears as a mass that involves the sphenoid and the skull base [1-3]. Although several cases of epignathus were reported previously, its incidence is one per 35,000 to one per 200,000 births [4]. Epignathus has a wide range of outcomes; the tumor size and resultant respiratory distress of the newborn are the most important factors affecting the prognosis and the management plan [5, 3, 22]. 

Polyhydramnios due to impaired fetal swallowing, intrauterine death due to fetal hydrops and pre-eclampsia are all acute presentations [3]. In the living delivered fetus, immediate management of the airway subsequent to delivery is an emergent procedure that can prevent death in a neonate. Thus, the antenatal diagnosis is essential [1, 3]. In previously reported cases, some extraordinary characteristics were presented, but none of them were too massive and huge. However, we will present a case of massive epignathus.

## Case presentation

**Figure 1:**
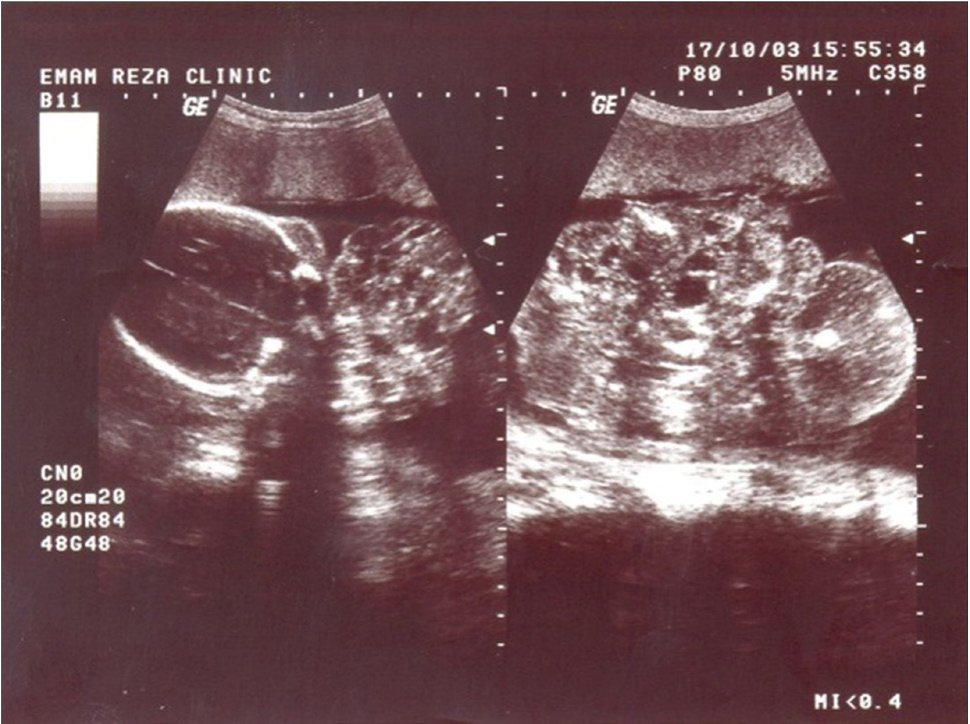
A heterogeneous, cystic-solid mass with calcified portions, anterior to the face.

**Figure 2:**
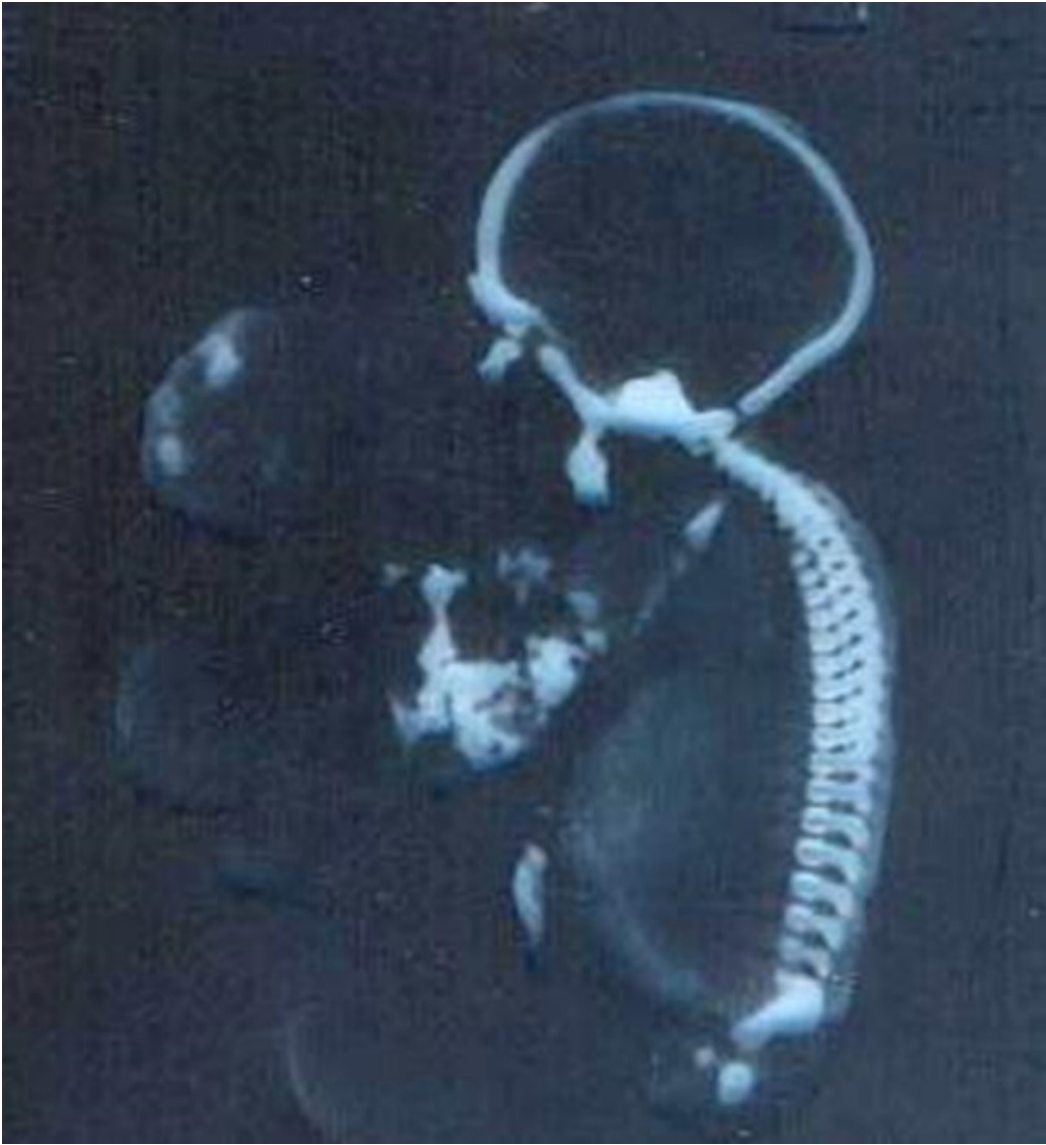
Calcified portions are apparent. Note the downward displaced mandible and extremely opened moth.

A female 35-year-old patient was referred to Imam Reza Hospital, Kermanshah, for the first time, to be evaluated by routine sonography. She did not have any previous systemic disease. She had two curettages two and four years ago because of abortions in the second and third months of pregnancies. The patient complained of mild vaginal discharge but denied any amniotic fluid leakage. Based on the sonography, the fetus was female, and it was in cephalic presentation. The fetus was alive, and the patient was 25 weeks pregnant. When assessing the head and face, a mass was observed in the anterior part of the face. There were also some echogenic spots with posterior shadows in some parts of the tumor (Figures 1-2).

**Figure 3:**
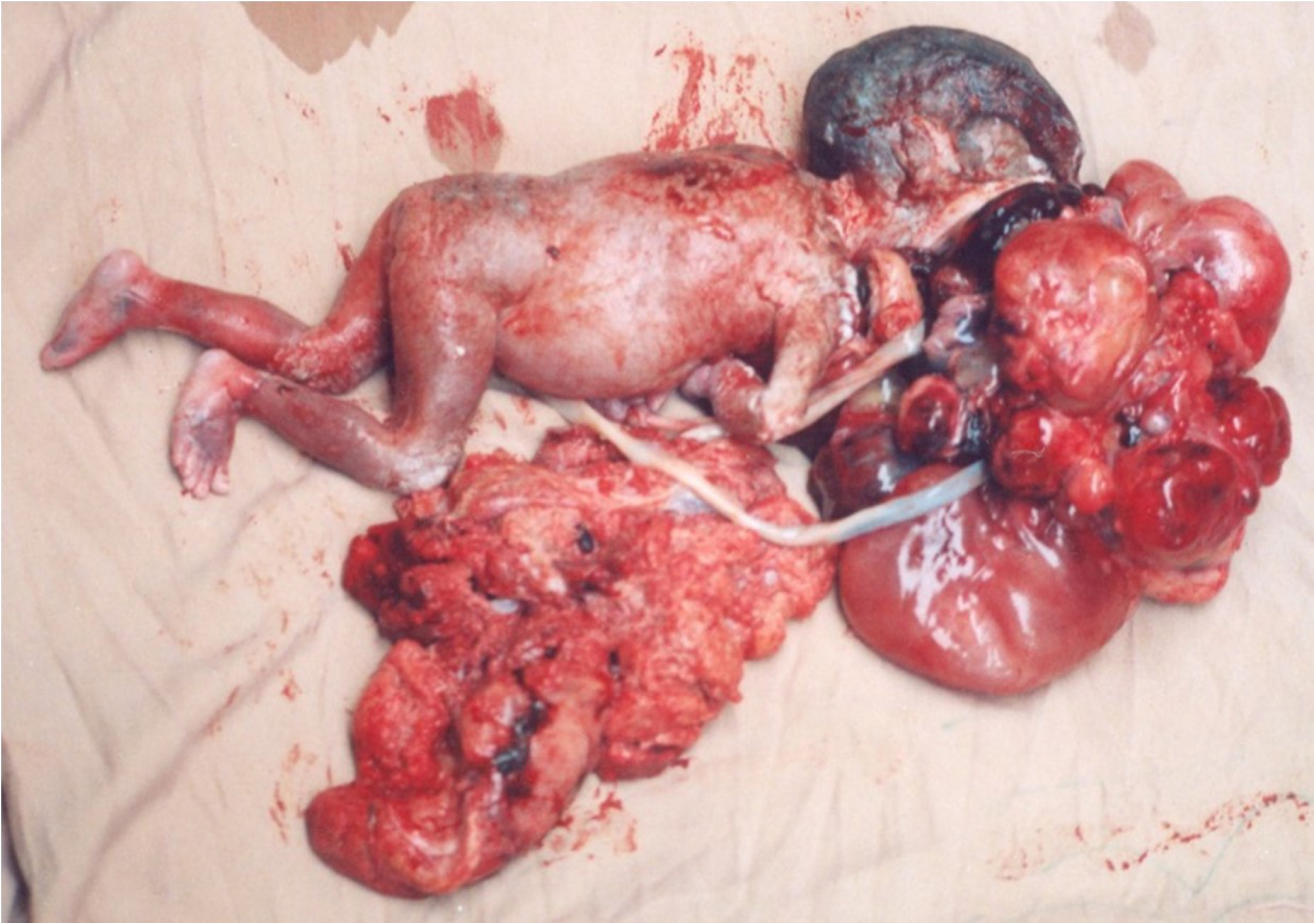
The aborted fetus.

Although the tumor was massive and it concealed the face, the main source of the tumor was not evident; nonetheless, the fetus’ mouth was wide open and it was supposed that the tumor is originating from the hard palate. No other anomaly was found in other parts of the body. The level of amniotic liquid was normal. No other apparent anomaly was reported regarding the rest parts of the body. Labor pain started in the 28th week of the pregnancy and the dead fetus was aborted. A large, 180 × 130 × 110 mm lobulated mass was extruded from the extremely opened mouth of the fetus (Figure 3). A pathologic sample was sent to the laboratory and benign mature teratoma was diagnosed.

## Discussion

Teratomas are rare tumors that may arise from different parts of the body. However, tumors arising at the level of the skull base are even rarer and are called epignathus [6]. Up to now, just less than 200 cases of this kind have been reported, and each one has had its specific characteristics and features. The sizes of reported teratomas are various; nevertheless, in our case, it was enormous. Tumor sizes found in other reports are presented in Table 1.

**Table 1:** Epignathus characteristics reported in some case reports.

**Author**	**Age of diagnosis**	**Sex**		**Size (mm)**
**Our case**	25 weeks	Female	Massive epignathus	200×130
**Sarioglu [6]**	23 weeks	Male	Epignathus combined with two fetus-like structures resembling acardius	119×77×93
**Clement [3]**	17 weeks	Male	Early diagnosis	40×30×25
**Gull [1]**	15 weeks	Male	Early diagnosis	70 ×70
**Hassan [9]**	At birth	Male	Not diagnosed	100×70×50
**Too [8]**	28 weeks	Male	Malignant	80×100
**Tsitouridis [10]**	29 weeks	Female	Epignathus originating from the hard palate	100×120
**Kotahari [14]**	At birth	Female	Not diagnosed	80×100
**Teixeira [15]**	15 weeks	Female	Treated by surgery	35×20×20
**Teixeira [15]**	24 weeks	Male	Treated by surgery	25×17
**Levine [16]**	At birth	Male	Treated by surgery	179×80×50
**Maeda [17]**	At birth	Female	Treated by surgery	27×22×10
**Shipp [18]**	29 weeks		Intracranial extension	100×70×40
**Johnston [19]**	At birth	Male	Intracranial extension and treated by surgery	40×50
**Yanez [7]**	21 weeks	Female	Massive epignathus	400×300
**Benson [20]**	19 weeks	Female	Treated by surgery	25×25
**Shah [21]**	30 weeks	Female	Large teratoma	150×120×100

The largest tumor was reported by Yanez et al., and it was 30 cm × 40 cm [7].

Epignathus can originate from face bones, especially the palate, sphenoid, and ethmoid; in some cases, the tumor is too big and comes out of the mouth [1]. This mass could lead to disorders in swallowing and it might increase the amniotic fluid. Epignathus has a wide range of outcomes. The tumor size is an important factor affecting the prognosis and the management plan [3-5], and our case had a considerable size. The fetus was dead at 28 weeks. In the case of small tumors, the neonate could survive via surgery and controlling the airways. 

Polyhydramnios was not observed in our case or Hassan’s case [9]. The likely cause of this phenomenon may be the preterm premature rupture of membranes (PPROM) in our case. According to the medical history, the patient had vaginal discharge, but amniotic fluid leakage was not noted, although paraclinical tests were not performed. In other reported cases, there were some degrees of polyhydramnios due to impaired fetal swallowing [6, 10]. In our case, the tumor was a benign teratoma, and it was similar to those reported by other studies [6, 11]. This type of tumor is usually benign, and its malignant variant is rarely reported [8].

Early diagnosis of the disease is possible, and some authors have reported this type of tumor even by the 15th week of pregnancy [1]. Since the tumor presented was very large, it was possible to diagnose the disease quicker if the case would have been referred earlier; nevertheless, it would not have any impact on the disease prognosis. Fetal teratomas have different types of differentiation, varying from tumors containing mature tissue like bones to immature teratoma [6]. Accurate sonography is needed to evaluate the anatomy of the fetus. Sonographic findings are often represented by a cystic mass with heterogeneous echogenicity and frequently echogenic foci due to calcified components, in front of the face or within the mouth. In addition, maternal serum alpha-fetoprotein may increase. Also, because of swallowing disorders of the fetus, polyhydramnios may be observed [1, 12]. Intrauterine death due to hydrops and pre-eclampsia are other forms of acute presentation [3, 11]. 

Teratomas have varied etiologies. Chromosomal abnormalities are associated with cases of congenital teratomas, which commonly include trisomy 13, ring X-chromosome, mosaicism with inactive ring X-chromosome, gonosomal pentasomy 49, gene mutations, or abnormalities in the early embryonic development [22]. The tumor is composed of at least two germ cell layers [6]. Epignathus is more common among females [13], and the fetus was female in our case, too. The origin of this tumor is not yet recognized. 

## Conclusion

Because the prognosis and management of epignathus depend on tumor size, early prenatal sonographic diagnosis is an important aspect in terms of follow-up and decision making.

## Conflict of Interest

The authors declare that there is no conflict of interest.
